# Increased Editorial Staff

**Published:** 1908-03

**Authors:** 


### EDITORIALS

The Business Office of the JOURNAL-RECORD is 1 1-2 to 5 1-2 South Broad St.
The Editorial Office is 1014-15 Century Bldg.
Address all Business Communications to Journal-Record of Medicine, 1 1-2 to 5 1-2 South Broad Street.
Make remittances payable to THE JOURNAL-RECORD OF MEDICINE.
On matters pertaining to the Editorial and Original communications, address Edgar G. Ballenger, M. D., Atlanta, Ga.
Reprints of original articles will be furnished at cost price. Requests for the same should always be made in THE MANUSCRIPT.
We will present, postpaid, on request, to each contributor of an original article, twenty (20) marked copies of THE JOURNAL-RECORD OF MEDICINE containing such article.

### INCREASED EDITORIAL STAFF.

In order to increase the value of the Journal-Record to our subscribers, it has been deemed expedient to increase our editorial staff, and beginning with this issue the augmented editorial fore becomes effective.

The prominence and experience of the men in charge of the various specialties of medicine will enable us to reflect the substantial advances and improvements, after a careful consideration of their real merit. Instead of having our collaborators furnish a few facts and observations, for each issue they are to give an ocasional digest of the subject, in which the points and discoveries recently advanced will be briefly and concisely presented. In this manner we believe the Journal-Record can supply its readers with the gist of the reliable medical literature, and in a form readily assimilable. The busy practitioner can thus obtain quickly knowledge that would otherwise be acquired at a great expenditure of time, or perhaps, not at all.

The chaff will, therefore, be separated from the grain, and erroneous or unreliable information kept from the man too busy to verify the claims by experiment or by more extensive reading.

This plan, of course, is not to supplant the original articles, but merely to supplement them. To read such a review or resume of the literature, however, in all probability will prove of considerably more value to the busy physician than would a nap over a long-winded original article upon some pet theory of the writer, perhaps without any practical application.

The idea we have sought to impress upon our collaborators is that preeminence should be given to the facts of practical value, for it is beyond the scope of our Journal to attempt to present ultra-scientific matter which properly belongs to a publication devoted exclusively to one subject, rather than to one mainly for the general practitioner, who, we are glad to admit, is the bone and sinew of our medical organization.

